# nQMaker: Estimating Time Nonreversible Amino Acid Substitution Models

**DOI:** 10.1093/sysbio/syac007

**Published:** 2022-02-09

**Authors:** Cuong Cao Dang, Bui Quang Minh, Hanon McShea, Joanna Masel, Jennifer Eleanor James, Le Sy Vinh, Robert Lanfear

**Affiliations:** Faculty of Information Technology, University of Engineering and Technology, Vietnam National University, 144 Xuan Thuy, Cau Giay, Hanoi 10000, Vietnam; Computational Phylogenomics Lab, School of Computing, Australian National University, Canberra, Australian Capital Territory 2601, Australia; Department of Earth System Science, School of Earth, Energy, and Environmental Sciences, Stanford University, Palo Alto, CA 94305, USA; Department of Ecology and Evolutionary Biology, University of Arizona, Tucson, AZ 85721, USA; Department of Ecology and Genetics, Plant Ecology and Evolution, Evolutionary Biology Center, Uppsala University, Uppsala, SE-752 36, Sweden; Faculty of Information Technology, University of Engineering and Technology, Vietnam National University, 144 Xuan Thuy, Cau Giay, Hanoi 10000, Vietnam; Division of Ecology and Evolution, Research School of Biology, Australian National University, Canberra, ACT 2601, Australia

## Abstract

Amino acid substitution models are a key component in phylogenetic analyses of protein sequences. All commonly used amino acid models available to date are time-reversible, an assumption designed for computational convenience but not for biological reality. Another significant downside to time-reversible models is that they do not allow inference of rooted trees without outgroups. In this article, we introduce a maximum likelihood approach nQMaker, an extension of the recently published QMaker method, that allows the estimation of time nonreversible amino acid substitution models and rooted phylogenetic trees from a set of protein sequence alignments. We show that the nonreversible models estimated with nQMaker are a much better fit to empirical alignments than pre-existing reversible models, across a wide range of data sets including mammals, birds, plants, fungi, and other taxa, and that the improvements in model fit scale with the size of the data set. Notably, for the recently published plant and bird trees, these nonreversible models correctly recovered the commonly estimated root placements with very high-statistical support without the need to use an outgroup. We provide nQMaker as an easy-to-use feature in the IQ-TREE software (http://www.iqtree.org), allowing users to estimate nonreversible models and rooted phylogenies from their own protein data sets. The data sets and scripts used in this article are available at https://doi.org/10.5061/dryad.3tx95x6hx. [amino acid sequence analyses; amino acid substitution models; maximum likelihood model estimation; nonreversible models; phylogenetic inference; reversible models.]

Amino acid substitution models play an essential role in model-based phylogenetic analyses of protein sequences. Amino acid substitutions are typically characterized by a time-continuous Markovian process, which is homogeneous, stationary, and reversible (Felsenstein 2003). Homogeneity means that the substitution rates remain constant during evolution; stationarity implies that the frequencies of the amino acids are at equilibrium; and reversibility indicates that substitution rates between any two amino acids are equal in both directions. Time-reversible models also obey detailed balance, that is, fluxes between any pair of amino acids have equal magnitude in both directions (Yang 2006).

Software such as FastMG (Dang et al. 2014) and QMaker (Minh et al. 2021) can estimate time-reversible models from collections of many multiple sequence alignments (MSAs). The empirically derived matrices of amino acid substitution rates are then typically fixed in phylogenetic analysis of protein sequences. Although mathematically and computationally convenient, there is empirical evidence that the assumption of time reversibility may be violated (Squartini and Arndt 2008; Naser-Khdour et al. 2019). The challenge has been in implementing software that is computationally efficient enough to estimate time nonreversible models. If nonreversible models are a better fit to the data than reversible models, we may see concomitant improvements in the estimation of tree topologies and branch lengths in phylogenetic analyses (the nonreversible models increase the number of free parameters in the inference).

Another benefit of nonreversible models is that they allow the root of a phylogenetic tree to be estimated in the absence of an outgroup (Bettisworth and Stamatakis 2021; Naser-Khdour et al. 2021). Rooting trees is an important part of studying evolutionary relationships among species. Unfortunately, the time-reversible models limit maximum likelihood (ML) methods to construct only unrooted trees because the likelihood of the tree remains the same regardless of the root position. To circumvent this limitation, most studies use outgroups or additional assumptions such as molecular clocks to root phylogenetic trees (Maddison et al. 1984; Huelsenbeck et al. 2002). However, finding an appropriate outgroup for the clade under study can still be a challenge in practice (Pearson et al. 2013). Other rooting methods include midpoint rooting (Farris 1972), minimal ancestor deviation (Tria et al. 2017), minimum variance rooting (Mai et al. 2017), using gene duplication (Iwabe et al. 1989), using indels (Lake et al. 2006), or using unrooted gene trees to root a species tree (Allman et al. 2011; Boussau et al. 2013). Nonreversible models remove the need for an outgroup because the root position is a parameter of the model, and different rooting positions will have different likelihoods. Recent studies based on simulated and empirical data have shown encouraging early results, demonstrating that nonreversible models can perform well on simulated data, and can give very similar results to outgroup rooting on empirical data (Bettisworth and Stamatakis 2021; Naser-Khdour et al. 2021).

We recently introduced QMaker (Minh et al. 2021), a software tool that allows users to efficiently estimate reversible models from large data sets. We showed that the algorithms in QMaker improve on existing methods (Whelan and Goldman 2001; Le and Gascuel 2008), and used QMaker to estimate a suite of new reversible matrices that can be applied to empirical data. QMaker uses a number of approaches to make it computationally feasible to rapidly estimate new Q matrices from large collections of empirical alignments but was restricted to estimating only time-reversible Q matrices.

In this article, we present nQMaker, which extends QMaker to allow the estimation of stationary nonreversible models from large collections of alignments. nQMaker combines a tree search strategy to determine rooted ML trees during the model estimation process and a ML algorithm to estimate 379 parameters of nonreversible models (instead of 189 parameters of reversible models) based on these rooted trees. We applied nQMaker to estimate six stationary nonreversible models: one from Pfam and five from clade-specific data sets for mammals, birds, insects, yeasts, and plants. Our results show that stationary nonreversible models not only improve the fit between the model and data, but also accurately infer rooted phylogenomic trees in those cases where we had confident *a priori* knowledge of the root position from other empirical analyses.

## Materials and Methods

### Estimating the Amino Acid Substitution Model

The amino acid substitution process is modeled by a time-homogeneous, time-continuous Markov process and represented by a $20 \times 20$ matrix $Q = \{q_{xy} \}$ where $q_{xy}$ is the number of substitutions between the two different amino acids $x$ and $y$ per time unit (diagonal values $q_{xx}$ are assigned such that the sum of all elements on row $x$ of $Q$ equals zero). In phylogenetic inference, the branch lengths reflect the number of substitutions per site, thus, the $Q$ matrix is normalized by dividing the factor $\mu$, where $\mu =-\sum \pi _x q_{xx}$, and $\pi _x$ is the equilibrium frequency of 20 amino acids.

The $Q$ matrix is used to calculate the transition probabilities between amino acids. Specifically, the so-called transition probability matrix $P\left( t \right) = \{p_{xy} \left( t \right)\}$, where $p_{xy} \left( t \right)$ is the probability of changing from amino acid $x$ to amino acid $y$ after $t$ substitutions, can be calculated as follows:

(1) \begin{align*}\label{eq1} P(t)=e^{Qt} \end{align*}

In a time-reversible model, the exchangeability rates between amino acid $x$ and amino acid $y$ are the same in both directions. We can only infer unrooted trees with time-reversible models because the likelihood of the tree remains the same regardless of the root placement (Felsenstein 1981). The reversible $Q$ matrix can be decomposed into a symmetric exchangeability rate matrix $R = \{r_{xy} \}$ and an amino acid frequency vector $\Pi = \{\pi _x \}$ such that $q_{xy} = \pi _y r_{xy}$ if $x \ne y$, otherwise, $q_{xx} = -\sum_y q_{xy}$. Thus, a reversible model consists of 208 free parameters (i.e., 189 parameters from the $R$ matrix and 19 parameters from $\Pi$ vector).

If the $Q$ matrix can be diagonalized, the matrix $P(t)$ is efficiently calculated as follows:

(2) \begin{align*}\label{eq2} P(t) = U\times e^{\Lambda t} \times U^{-1} \end{align*}

where $\boldsymbol\Lambda$ is the diagonal matrix of eigenvalues of $Q$; $U$ is the matrix of eigenvectors of $Q$ and $U^{-1}$ is its inverse matrix.

In this article, we relax the assumption of time-reversibility by removing the symmetric constraint of the $R$ matrix. Therefore, we need to estimate all off-diagonal elements of the $Q$ matrix. This increases the number of free parameters from 208 to 379. The transition probability matrix $P\left( t \right)$ can be calculated using a combination of eigen-decomposition and scaling-squaring techniques provided by the Eigen3 library (Guennebaud and Jacob 2010), which is already incorporated in IQ-TREE 2 (Minh et al. 2020). Specifically, IQ-TREE 2 uses eigen-decomposition to diagonalize $Q$ into its (complex) eigenvalues, eigenvectors, and inverse eigenvectors to calculate $P(t)$ using Equation (2). If $Q$ is not diagonalizable, then IQ-TREE 2 employs the scaling-squaring technique to compute $P(t)$ based on the second-order Taylor expansion of Equation (1).

Given a data set ${{\bf D}} = \{D_1,\ldots,D_n \}$ consisting of $n$ multiple amino acid sequence alignments, let ${{\bf T}} = \{T_1,\ldots T_n\}$ be the tree set corresponding to the data set **D**, that is, $T_i$ is the ML tree of alignment $D_i$. The ML estimation method determines the tree set **T** and a model $Q$ to maximize the likelihood value $L\left( {Q, {{\bf T}};{{\bf D}}} \right)$. We assume that amino acid substitutions among alignments and sites are independent, thus, the likelihood value $L\left( {Q, {{\bf T}};{{\bf D}}} \right)$ can be calculated as follows:

(3) \begin{align*}\label{eq3} L\left( {Q, {{\bf T}};{{\bf D}}} \right) & = \prod_{i= 1}^n L(Q, T_{\rm i} ;D_i )\nonumber\\ & = \prod_{i = 1}^n \prod_{j = 1}^{l_i } L\left( {Q, T_{\rm i} ;D_{ij} } \right) = \prod_{i = 1}^n \prod_{j = 1}^{l_i} P(D_{ij} \vert Q, T_{\rm i}) \end{align*}

where $l_i$ is the length of alignment $D_i$ and $D_{ij}$ is the data at site $j$ of alignment $D_i$. The likelihood value $L(Q, T_{\rm i} ;D_{ij})$ can be calculated by the conditional probability $P(D_{ij} \vert Q, T_{\rm i} )$ of data $D_{ij}$ given the model $Q$ and the tree $T_i$.

As amino acid substitution rates vary among sites, we incorporate the site rate heterogeneity by determining site rate models ${\rm V} = \{V_1,\ldots,V_n \}$ for alignments **D**, that is, $V_i$ is the site rate model of alignment $D_i$. Typically, a site rate model combines a $\Gamma$ distribution of rates, a proportion of invariant sites (Yang 1993; Gu et al. 1995), or a distribution-free rate models (Yang 1995). The best-fit rate model for each MSA or locus was determined by using ModelFinder (Kalyaanamoorthy et al. 2017). The likelihood value $L\left( {Q, {{\bf T}}, {{\bf V}};{{\bf D}}} \right)$ is now technically calculated as follows:

(4) \begin{align*}\label{eq4} L\left( {Q, {{\bf T}}, {{\bf V}};{{\bf D}}} \right) &= \prod_{i = 1}^n \prod_{j = 1}^{l_i } L\left( {Q, T_{\rm i}, V_i ;D_{ij} } \right)\nonumber\\ & = \prod_{i = 1}^n \prod_{j = 1}^{l_i } P\left( {D_{ij} \vert Q, T_{\rm i} , V_i } \right) \end{align*}

where $P(D_{ij} \vert Q, T_{\rm i}, V_i )$ is the conditional probability of data $D_{ij}$ given the model $Q,$ the tree $T_i$, and the site rate model $V_i$.

The ML estimation method determines the parameters of model $Q$, the trees **T**, and the site rate models **V** to optimize the likelihood value $L\left( {Q, {{\bf T}}, {{\bf V}};{{\bf D}}} \right)$ in Equation (4).

### Using nQMaker to Estimate Time Nonreversible Models

Estimating the $Q$ matrix is computationally difficult because we have to simultaneously estimate its parameters, the trees **T**, and the site rate models **V**. A number of approximate maximum-likelihood methods have been proposed to estimate model $Q$ from large data sets (Whelan and Goldman 2001; Le and Gascuel 2008; Dang et al. 2014; Minh et al. 2021). The methods show that the parameters of $Q$ can be accurately estimated using nearly optimal trees **T** and site rate models **V**. Thus, we can iteratively estimate the model $Q$, the trees **T**, and site rate models **V** to optimize the likelihood value $L\left( {Q, {{\bf T}}, {{\bf V}};{\rm {\bf D}}} \right)$. Most recently, QMaker (Minh et al. 2021) has been shown to efficiently estimate reversible models using this approach.

**Figure 1.**
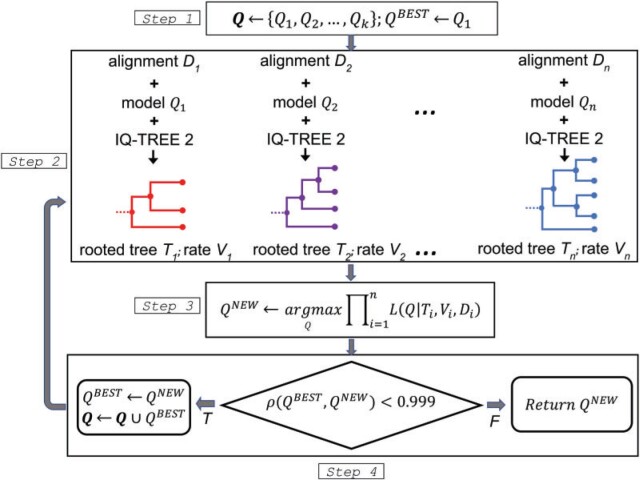
The flowchart of nQMaker to estimate a time nonreversible model from a collection of multiple protein sequence alignments.

The nQMaker approach presented here extends QMaker to estimate nonreversible models from large data sets of MSAs. It is composed of four main steps as illustrated in Figure 1 and described as follows:

1. Initialize a set of candidate matrices **Q**; typically we use LG (Le and Gascuel 2008), JTT (Jones et al. 1992), and WAG (Whelan and Goldman 2001) as three initial matrices. Set the current best matrix $Q^{BEST} :=\ LG$.
2. For each $D_i$, determine $Q_i \in {{\bf Q}}$ as the best-fit matrix, $V_i$ as the best site rate model, then employ IQ-TREE 2 to estimate an ML tree $T_i$ based on $Q_i$ and $V_i$ (if $Q_i$ is nonreversible $T_i$ is a rooted tree). Let $\mathcal{T}_i$ and $\mathcal{L}_i$ be the topololgy and branch lengths of tree $T_i$, respectively. For clade-specific data sets, instead of constructing a separate topology $\mathcal{T}_i$ for each locus, we estimate only one edge-linked topology $\mathcal{T}$ across all loci, although allowing rate variation across all loci using and edge-linked partitioned model.
3. With $V_i$ and $\mathcal{T}_i$ fixed, estimate $Q^{NEW}$ and $\mathcal{L}_i$ to maximize the log-likelihood function. Precisely, we iterate two substeps:
  a) With $V_i, \mathcal{T}_i$, and $\mathcal{L}_i$ fixed, estimate $Q^{NEW}$.
  b) With $V_i, \mathcal{T}_i$, and $Q^{NEW}$fixed, estimate $\mathcal{L}_i$. If the log-likelihood is increased more than 0.1 go to sub step a, otherwise, go to the next step 4.
4. Assign $Q^{BEST} := Q^{NEW}$. If the Pearson correlation coefficient between $Q^{BEST}$ and $Q^{NEW}$ is less than $0.999$, add $Q^{BEST}$ to the set of candidate matrices **Q**, repeat from step 2. Otherwise, return $Q^{BEST}$ as the final matrix for the database ${{\bf D}}$.

The key difference between nQMaker and QMaker is that nQMaker uses rooted ML trees to estimate the 379 parameters of nonreversible models, rather than using unrooted trees to estimate the 189 parameters of reversible models in QMaker. Experiments on large data sets show that the estimation process usually stops after three iterations.

### Data Sets

We used the general Pfam database (seed alignments version 31) and the same five clade-specific data sets as used in the QMaker paper (i.e., Plant, Bird, Mammal, Insect, and Yeast). The Pfam data set consists of 13,308 MSAs from 1,150,099 sequences including 3,433,343 sites. The Pfam data set was randomly divided into training and testing sets each containing 6654 MSAs. The clade-specific data sets contain between 1308 (Plant) and 7295 (Bird) loci, and between 38 (Plant) and 343 (Yeast) sequences. For each clade-specific data set, we randomly selected 1000 MSAs for estimating a nonreversible model and used the remaining MSAs for testing the estimated model. We filtered out small loci with fewer than 50 sites in the Insect data set (no other data sets contained loci with fewer than 50 sites).

The six data sets are summarized in Table 1 and available from the Supplementary material available on Dryad at https://doi.org/10.5061/dryad.3tx95x6hx.

**Table 1.** Summary of six data sets used for training and testing nonreversible models

Data set	No. of sequences	No. of sites	Training	Testing	References
Pfam	1,150,099	3,433,343	6654	6654	El-Gebali et al. (2018)
Bird	52	4,519,041	1000	6295	Jarvis et al. (2015)
Insect	144	595,033	1000	1482	Misof et al. (2014)
Mammal	90	3,050,199	1000	3162	Wu et al. (2018)
Plant	38	432,014	1000	308	Ran et al. (2018)
Yeast	343	1,162,805	1000	1408	Shen et al. (2018)

Many genome annotations are contaminated with Pfams that do not belong to the ostensibly sequenced and assembled species’ genome but to one of its parasites (Breitwieser et al. 2019; Salzberg 2019). Therefore, we excluded all Pfam domains whose annotations suggested parasitic origin, for example, “viral” or “transcriptase” (James et al. 2021) from our training and testing Pfam sets to create a cleaned training Pfam set of 3655 MSAs and a cleaned testing Pfam set of 3611 MSAs. We then estimated a new nonreversible model from this cleaned Pfam data set, which we call NQ.cPfam. In the following, we primarily consider the full Pfam data set as our Pfam model.

### Model Estimation

We used nQMaker to estimate nonreversible models (denoted NQ) from the training sets of six data sets, that is, NQ.pfam for Pfam, NQ.cPfam for cleaned Pfam, NQ.plant for Plant, NQ.bird for Bird, NQ.insect for Insect, NQ.mammal for Mammal, and NQ.yeast for Yeast. The reversible models for the data sets (Q.pfam, Q.plant, Q.bird, Q.insect, Q.mammal, and Q.yeast) were obtained from the QMaker paper (Minh et al. 2021). We compared nonreversible models and reversible models on testing sets using Bayesian information criterion (BIC) values (Schwarz 1978). All models were tested with rate models “$+$G4” ($\Gamma$ distribution with four categories), “$+$I” (invariant site model), and “$+$R$c$” (distribution-free rate model with $c$ categories). The reversible models were also tested with “$+$F” option (i.e., amino acid frequencies were directly estimated from testing data). Note that each nonreversible model is represented by a single matrix $Q$, therefore “$+$F” option is not valid for nonreversible models.

The nonreversible model for the Pfam data set was estimated with two commands in IQ-TREE 2: where $\texttt{-S ALN_DIR}$ option specifies the directory of training data; $\texttt{-mset LG,WAG,JTT}$ option defines the initial candidate matrices to reduce computational burden; $\texttt{-cmax 4}$ option restricts up to four categories for the rate heterogeneity across sites. The first command outputs the best models to $\texttt{ALN_DIR.best_model.nex}$ and the best trees to $\texttt{ALN_DIR.treefile}$. These files are then used as the input for the second command, which estimates a joint nonreversible Q matrix across all input alignments.

$$\texttt{iqtree2 -S ALN_DIR -mset LG,WAG, JTT -cmax 4}$$

$$\texttt{iqtree2 -S ALN_DIR.best_model.nex -te ALN_DIR.treefile --model-joint NONREV+FO}$$

For clade-specific data sets, we used $\texttt{-p}$ option instead of $\texttt{-S}$ option to estimate an edge-linked partition model with a single tree topology shared across all loci. This $\texttt{-p}$ option is typically used for the estimation of trees using concatenated sequences, assuming a single species tree but rescaling the branch lengths of the individual single-locus trees. Previous work has shown that edge-linked partitioned models usually perform best among a range of related options (Duchêne et al. 2019).

### Performance Comparison

We compared the nonreversible (NQ) and reversible (Q) models on the test alignments of the Pfam, bird, mammal, insect, plant, and yeast data sets. For each data set, we counted the number of test alignments for which the NQ model was a better fit to the data than the Q model using the BIC and BIC weight (Schwarz 1978).

To ask whether the improvement in fit of nonreversible models is associated the length of an alignment, we analyzed both single-locus and concatenated alignments. We first assessed the relationship between single-locus alignment length on the relative model fit of NQ models using our five clade-specific data sets. For each clade-specific data set, we classified the test alignments into 10 bins according to their length, then calculated the Spearman correlation between the rank of the bin and the proportion of alignments which were best fit by the NQ model for that data set. We also examined the fit of the new NQ models on longer concatenated alignments. To do this, we assessed the model fit of NQ models on concatenated alignments from clade-specific data sets with 1, 5, 10, 20, 50, 100, and 200 loci. For each number of loci, we randomly created 100 replicate concatenated alignments, then calculated the proportion of 100 replicates where the NQ model was a better fit to the data than the Q model. For example, for the Plant data set and the case of 10 loci, we created 100 concatenated alignments each composed of 10 different randomly selected loci from the Plant test data set, then compared the fit of NQ.plant to Q.plant on those 100 concatenated alignments.

We then tested whether the six new nonreversible matrices affect tree topology inference (we consider the seventh model, NQ.cPfam, later). For each single-locus MSA in each test data set, we inferred an unrooted ML tree using the best-fit model among nine published reversible models (JTT, WAG, LG, Q.pfam, Q.plant, Q.mammal, Q.bird, Q.insect, and Q.yeast), which we call T$_{\rm REV}$. We then performed a second IQ-TREE run considering 15 models, comprising the same nine reversible models but adding six new nonreversible models (NQ.pfam, NQ.plant, NQ.mammal, NQ.bird, NQ.insect, or NQ.yeast), to infer another tree T$_{\rm NEW}$. If one of the six NQ models fits the data better, then T$_{\rm NEW}$ will be rooted and will therefore differ from T$_{\rm REV}$. In this case we launch another IQ-TREE run with the same matrix as T$_{\rm REV}$ but using a different random seed. We call the resulting tree T$_{\rm REV2}$. Otherwise, if the NQ models do not provide a better fit, then the 2$^{\rm nd}$ run will use the same model as the first run but T$_{\rm NEW}$ might still be different from T$_{\rm REV}$ due to search heuristics. Thus, for each alignment we now have three trees T$_{\rm REV}$, T$_{\rm NEW}$, and T$_{\rm REV2}$ when a nonreversible model fits the data best.

We then compared the three trees for each alignment when a nonreversible model fits the data best using normalized Robinson–Foulds (nRF) distances (Robinson and Foulds 1981). The nRF distance simply normalizes the standard RF distance (the number of splits by which two trees differ) by dividing it by the maximum possible distance between those two trees. Thus, a value of 0 indicates two identical trees, and a value of 1 indicates two trees that are maximally different, that is, share no splits in common. To calculate the nRF we first unrooted the rooted tree (if required) then used IQ-TREE to calculate the nRF with options $\texttt{-rf1 ---normalize-dist}$. To ask whether nonreversible models lead to bigger changes in tree topologies than expected from search heuristics alone, we compared the two distributions of nRF distances: nRF(T$_{\rm NEW}$, T$_{\rm REV})$, which is the distribution of differences driven by a nonreversible model being a better fit to the data than a reversible model; and nRF(T$_{\rm REV}$, T$_{\rm REV2})$, which is the distribution of differences driven by changing the random number seed under a reversible model. If nonreversible models have an appreciable effect on tree topologies, we would expect nRF(T$_{\rm NEW}$, T$_{\rm REV})$ to be composed of larger differences than nRF(T$_{\rm REV}$, T$_{\rm REV2})$.

We compared NQ.Pfam to NQ.cPfam to ask specifically whether cleaning the Pfam data set has any measurably impact on the Q matrix or the model performance. To do this, we measured the BIC score of both models on the test MSAs from both the Pfam data set and the cleaned Pfam data set (cPfam).

## Results

### Nonreversible Models Generally Provided much Better Fit to the Data than Reversible Models

We used the training data of the Pfam, bird, mammal, insect, plant, and yeast data sets to estimate nonreversible models (NQ) and compare them with the reversible models on the test alignments. For each data set, we counted the number of test alignments for which the NQ model was better than the Q model using the BIC. Table 2 shows that the NQ models fit the data better than the Q models for all clade-specific data sets, typically being selected as the best fit model for 60–70$\%$ of the test alignments. However, for the Pfam data set the reversible model Q.pfam outperformed the nonreversible model NQ.pfam, with the former being the best fit for two-thirds of the test alignments.

**Table 2.** The number of alignments where the NQ and Q models were selected as best-fit on six data sets

	Pfam	Bird	Insect	Mammal	Plant	Yeast
$NQ$	2218	3895	1001	1950	190	869
	(33.33$\%$)	(61.87$\%$)	(67.54$\%$)	(61.67$\%$)	(61.69$\%$)	(61.72$\%$)
$Q$	4436	2400	481	1212	118	539
	(66.67$\%$)	(38.13$\%$)	(32.46$\%$)	(38.33$\%$)	(38.31$\%$)	(38.28$\%$)

*Note*: For example, the NQ model outperformed the Q model on 61.87$\%$ of testing alignments in the Bird data set.

We suspected that the poor performance of NQ.pfam might be caused by a large number of small Pfam alignments (76$\%$ of Pfam test alignments have $\le 100$ sequences). This is supported by post hoc data analysis, which shows that the NQ.pfam model outperformed the Q.pfam model in just 26$\%$ of small test alignments (with $\le 100$ sequences) but in 56$\%$ of large test alignments (with $> 100$ sequences). The median size of alignments best fit by NQ.pfam (78 sequences) is much larger than the median size of alignments best fit by Q.pfam (26 sequences). We further examined the effect of the number of sequences in the alignment on the model fit of NQ.pfam by classifying test alignments in Pfam into 10 subsets (bins) by the number of sequences such that $i^{\rm th} (i = 0 \ldots 9)$ bin contains all test alignments with $\left( {i \times 100 + 1} \right)$ to $(i \times 100 + 100)$ sequences. We calculated the Spearman correlation between the rank of the bin and the proportion of alignments in the bin which are best fit by NQ.pfam. The Spearman correlation value is 0.903 indicating that the model fit of NQ.pfam increases with the number of sequences in testing alignments.

**Figure 2.**
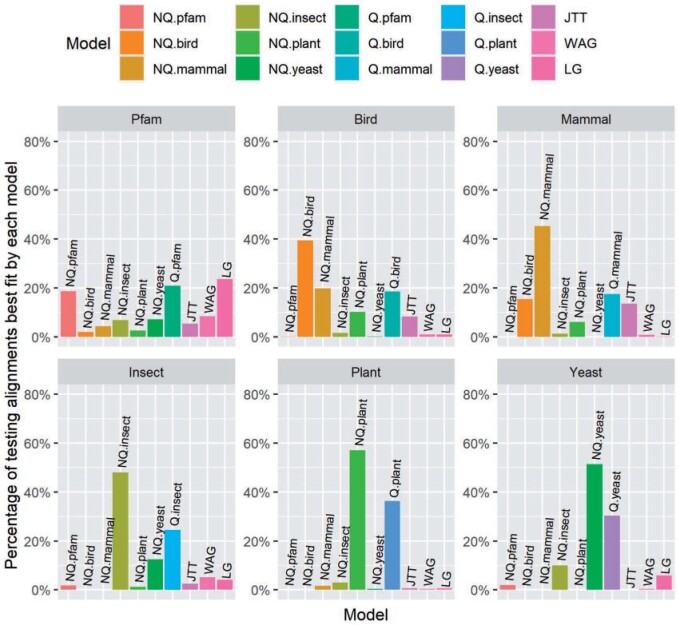
The percentage of testing alignments best fit by each model in Pfam and five clade-specific data sets.

Second, we compared 10 different models comprised of the 6 nonreversible models, 3 general models (JTT, LG, and WAG), and 1 best-fit reversible model for each testing data set (e.g., Q.pfam for Pfam or Q.plant for Plant). Similar to the results above, these results show that the nonreversible models performed best for the clade-specific data sets, but not for the Pfam data set (Figure 2). In most cases, the second best model for each clade-specific data set was the reversible model previously estimated for that data set (e.g., Q.mammal is the second best data set behind NQ.mammal for the mammal data set, Figure 2).

We also used the BIC weights across loci to measure the fit of each MSA/locus with 15 models (6 NQ models, 6 Q models, JTT, LG, and WAG). The distributions of BIC weights across loci for six test data set sets (Fig. S1 of the Supplementary material available on Dryad) show similar findings as above: the clade-specific nonreversible models perform best for the clade-specific data sets, and the reversible Pfam model (Q.Pfam) performs best for the Pfam data set, with the nonreversible model (NQ.Pfam) being second-best.

Finally we asked whether cleaning the Pfam data set improved performance, by comparing the NQ.Pfam model to the NQ.cPfam model estimated from the cleaned training Pfam set (cPfam; see Material and Methods), by comparing their performance on the test MSAs from both the Pfam and cPfam data sets. NQ.Pfam performed better than NQ.cPfam both data sets: it had a lower BIC score than the NQ.Pfam on 2519 (69.7$\%$) out of the 3611 cPfam test MSAs, and on 4774 (71.7$\%$) out of 6654 Pfam test MSAs. Thus, the contaminated MSAs in the Pfam data set did not adversely affect the quality of the NQ.pfam model.

### Nonreversible Model Fit Correlates with Alignment Lengths

Analyses comparing the length of single-locus MSAs to the proportion of MSAs best fit by a nonreversible model showed variable results among data sets. The Spearman correlations were 0.47 for NQ.Bird, 0.87 for NQ.insect, 0.56 for NQ.Mammal, $-$0.02 for NQ.Plant, and 0.42 for NQ.yeast. This suggests that both the strength and the sign of the correlation between alignment length and the relative fit of nonreversible models can vary considerably, depending on the data set.

**Figure 3.**
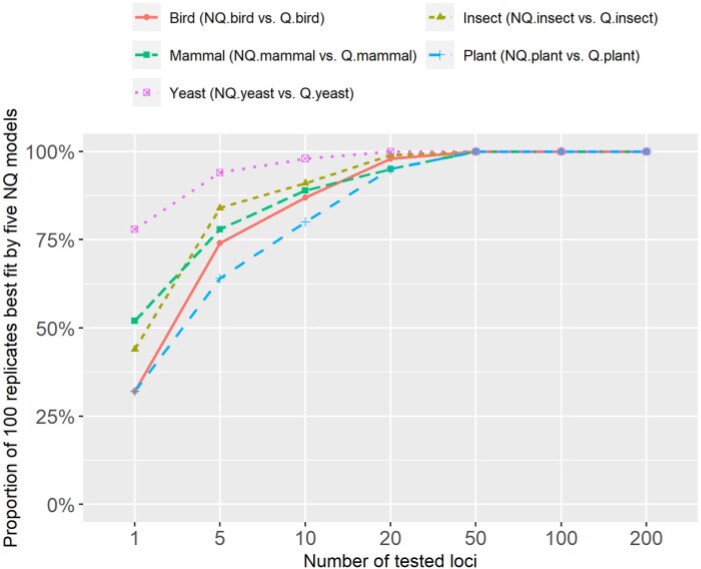
The proportion of 100 concatenated alignments best fit by nonreversible models on five clade-specific data sets.

We next sought to examine the relative fit of the new NQ models and alignment length using much longer concatenated alignments. The results on five clade-specific data sets (see Figure 3) show that the proportion of replicates for which the NQ model is the best-fit model increases with the number of loci in the concatenated alignment. The NQ models outperformed the corresponding Q models on almost all concatenated alignments with $\ge 20$ loci, and on practically all concatenated alignments with $>$50 loci (Fig. 3). The difference in BIC scores between Q and NQ models increased linearly with the number of loci for all five clade-specific data sets. We note that for alignments with fewer than 20 loci, the relative fit of the NQ models varied among replicates, which we hypothesize is related to stochasticity in the amount of phylogenetic information contained in short alignments. This result suggests that for phylogenomic data sets with many loci, nonreversible models will almost always outperform reversible models in terms of their model fit, and may therefore lead to more accurate estimation of trees and branch lengths in these cases.

### Analysis of the Properties of Nonreversible Models

**Figure 4.**
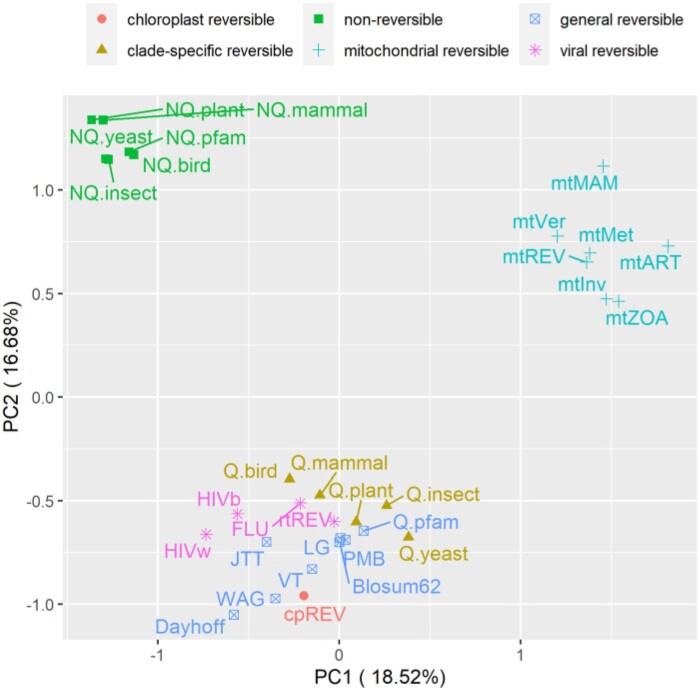
Principal component analysis of 6 nonreversible models and 25 reversible models. Each model was represented by one vector of all (400) elements of the Q matrix. The nonreversible models are grouped into one distinct cluster.

**Figure 5.**
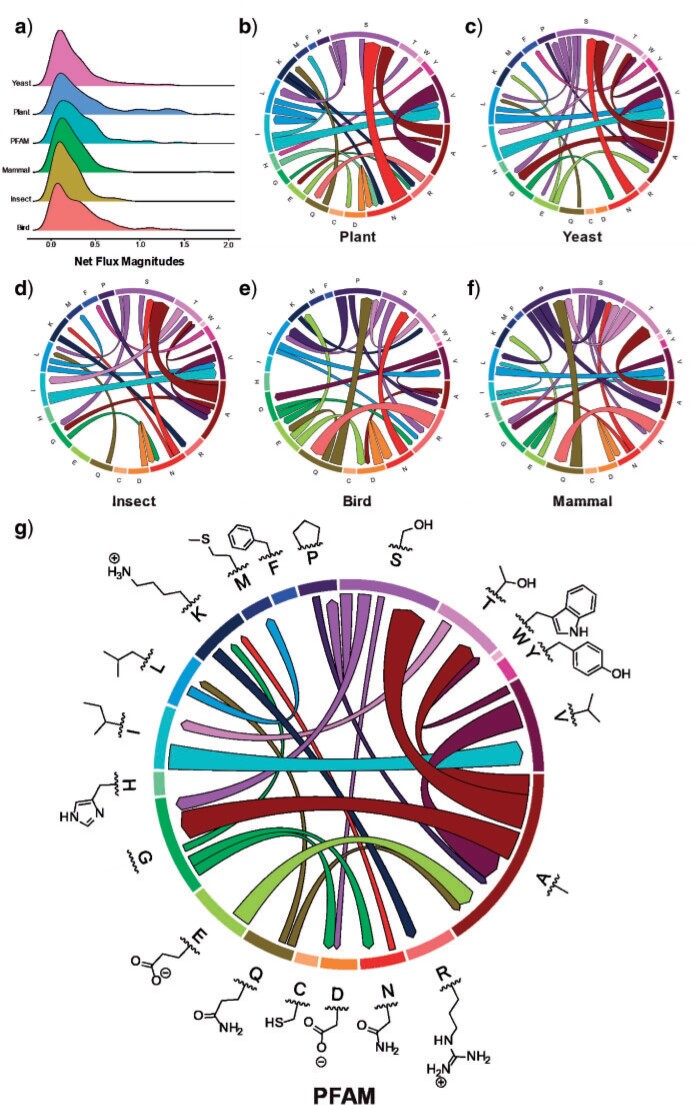
Departures from reversibility are large, and vary across taxonomic groups. Net fluxes are calculated from nonreversible rate matrices as $net\ flux_{ij} = \vert flux_{i \to j } - flux_{j \to i} \vert = \vert (rate_{i \to j} \times freq_i ) - (rate_{j \to i} \times freq_j )\vert$. a) The smoothed histograms (calculated by kernel density estimation with R package ggridges) show each taxonomic group’s distribution of net flux magnitudes across all amino acid pairs, normalized for each pair relative to net flux as $(2 \times net flux_{ij} ) / (flux_{i \to j} + flux_{j \to i} )$. b–g) Chord diagrams show the largest 5$\%$ of net fluxes between pairs, that is, most information about net flux magnitude is given by presence versus absence in the chord diagrams. The size of each band along the outer circle represents the equilibrium frequency of each amino acid, and the width of each chord at its attachment points is proportional to the magnitude of net flux between each pair of amino acids for that taxonomic group. Color in chord diagrams is for ease of interpretation and contains no extra information.

We used principal component analysis (PCA) to visualize the difference between nonreversible and reversible models. Each model was represented by one vector of all amino acid substitution rates and subsequently analyzed by our R script (available at https://doi.org/10.5061/dryad.3tx95x6hx). Figure 4 illustrates the PCA analysis of 6 nonreversible models and 25 existing reversible models. Figure 4 shows that the models group into three distinct clusters, that is, one cluster of nonreversible models, one cluster of reversible models estimated from mitochondrial data, and another cluster of reversible models estimate from other genomic regions. This PCA analysis indicates that nonreversible models provided a very distinct pattern of amino acid substitutions not captured by existing reversible models. To understand these NQ matrix substitution patterns, we calculated the net flux between each amino acid pair for each clade. Figure 5 shows drastic departures from reversibility in all taxonomic groups, and substantial differences among taxonomic groups. The largest nonreversible fluxes are not between particularly codon-adjacent or (what are typically considered) chemically similar amino acids. Further study is needed to understand the contributions of amino acid chemistry to the direction and magnitude of the fluxes, and thus to the nonreversible evolutionary process summarized in the NQ matrices.

### Nonreversible Models Correctly Inferred the Root Placement of Reconstructed Trees

We assessed the root placement of trees reconstructed with nonreversible models from the two clade-specific data sets where previous publications have indicated a well-supported root placement, that is, the plant tree from Ran et al. (2018) and the bird tree from Jarvis et al. (2015). The branches on reconstructed trees were labeled with rootstrap values calculated from an ultrafast bootstrap analysis (Hoang et al. 2017), that is, the rootstrap value for a branch is defined as the fraction of rooted bootstrap trees which have the root on that branch (Naser-Khdour et al. 2021). We also performed approximately unbiased (AU) test (Shimodaira 2002) with 1000 replicates for all branches to determine a confidence set of root branches (i.e., branches with $p_{AU} > 0.05$ are considered as potential root branches and included into the confidence set) (Naser-Khdour et al. 2021).

**Figure 6.**
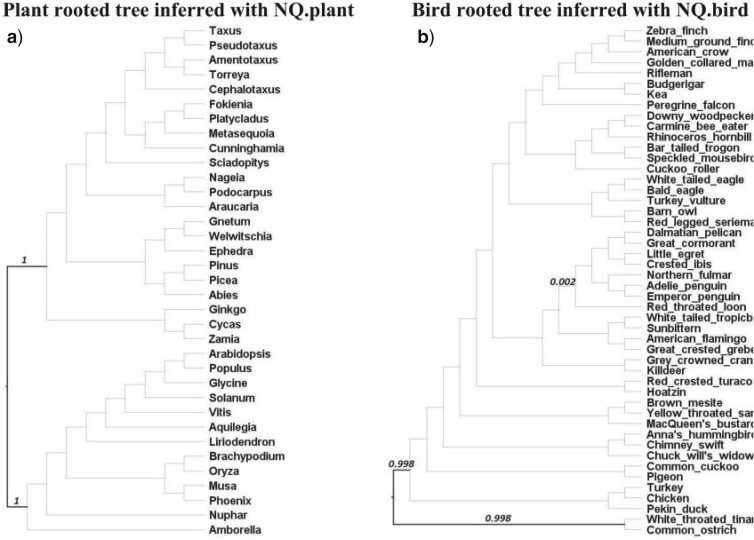
The plant-rooted tree of 35 species (a) reconstructed from a concatenated protein alignment of 1308 loci using IQ-TREE 2 with the NQ.plant model. The bird-rooted tree of 48 species (b) reconstructed from a concatenated protein alignment of 8295 loci using the NQ.bird model. Bold branches are branches contained in the confidence set of the AU test, numbers displaying on branches are the rootstrap values greater than zero.

Figure 6 illustrates the plant-rooted tree and the bird-rooted tree reconstructed using NQ.plant and NQ.bird, respectively. The expected root branch, based on the analysis of the plant tree (Ran et al. 2018) using outgroups, belongs to the AU test confidence set and has a rootstrap value of 1.000 (supported by all bootstrap trees). Similarly, the expected root branch, based on previous analyses of bird tree (Jarvis et al. 2014) using outgroups, was confirmed by the AU test and labeled with a very high-rootstrap value of 0.998. These results demonstrate that nonreversible models reconstructed rooted trees with high confidence in root placements that agree with the roots inferred by outgroup rooting.

### Nonreversible Models Inferred Different Locus Trees and Coalescent-based Species Trees

**Figure 7.**
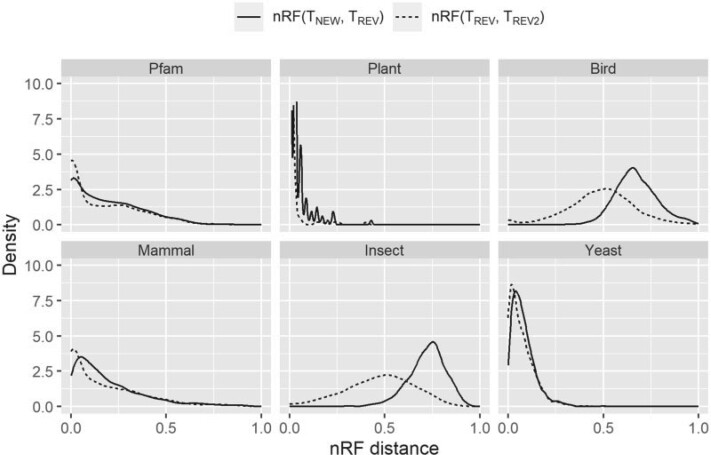
Distributions of normalized Robinson–Foulds (nRF) distances between the trees inferred by nonreversible and reversible models. The solid line is the distribution where the best-fit model is one of the new nonreversible models inferred in this study (NQ.pfam, NQ.plant, NQ.mammal, NQ.bird, NQ.insect, or NQ.yeast). Comparing to best-fit reversible model, new model shows an effect on the tree topology (the best-fit reversible model is chosen from nine existing models Q.pfam, Q.plant, Q.mammal, Q.bird, Q.insect, Q.yeast, LG, JTT, or WAG; and is showed by the dotted line).

We next examined whether nonreversible models affect the topologies of estimated ML trees from single loci. The two nRF distributions are depicted in Figure 7. We found that using nonreversible models changes locus tree topologies in every data set (the solid line) and in many data sets, the extent of topological changes induced by the nonreversible models is larger substantially greater than those induced by changing the random number seed with otherwise identical reversible model analyses (the dotted line).

**Figure 8.**
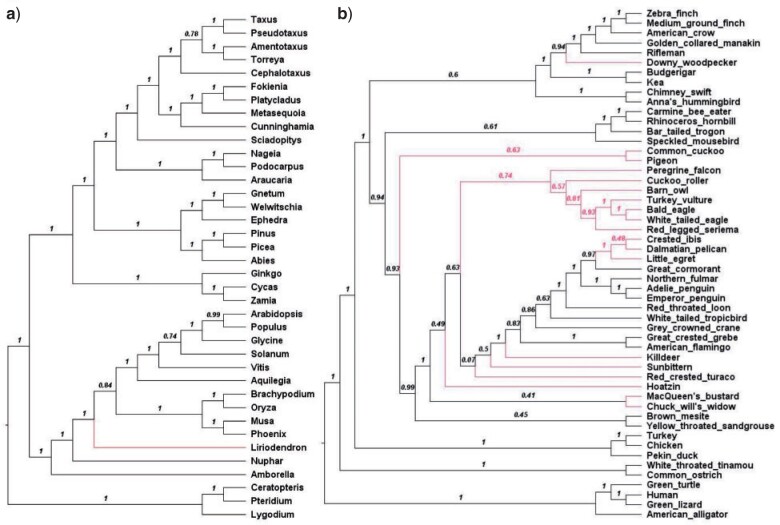
ASTRAL$_{\rm NEW}$ species trees from plant (a) and bird (b) data reconstructed from the set of T$_{\rm NEW}$ locus trees. Shown on each internal branch the ASTRAL local posterior probability.

Because of the observed differences between gene tree topologies, we examined to what extent it influences the reconstruction of species trees using coalescent-based methods. These methods use distributions of single-locus trees to infer a species tree, so changes in the underlying single-locus trees may affect species-tree inference. To this end, for each clade-specific data set, we used ASTRAL version 5.15 (Zhang et al. 2018) to construct a species tree ASTRAL$_{\rm REV}$ from the set of trees estimated using reversible models (T$_{\rm REV})$ and a species tree ASTRAL$_{\rm NEW}$ from the set of T$_{\rm NEW}$ trees, estimated using the best-fit models regardless of whether they were reversible or nonreversible For plant data set, the ASTRAL$_{\rm REV}$ tree and the ASTRAL$_{\rm NEW}$ tree (Figure 8a) differ by the position of a single taxon, Liriodendron. The topological differences are more pronounced for Mammals, Insects, Yeasts, Birds with 2, 10, 15, and 17 different branches between the ASTRAL$_{\rm REV}$ and ASTRAL$_{\rm NEW}$ trees. Figure 8b highlights these differences for the Bird data set, the other trees are available as Supplementary material available on Dryad.

## Discussion

Most phylogenetic analyses of protein sequences use time-reversible substitution models, which can be limited in their ability to accurately model the biological process of amino acid substitution. Although estimating time nonreversible models is complicated and computationally expensive (e.g., 1.5 days with a computer of 36 cores for estimating NQ.plant and 105 days with the same computer for estimating NQ.pfam), it has the potential to allow models of sequence evolution to better reflect the underlying evolutionary mechanisms, and hence could improve the estimation of evolutionary relationships and timescales among species.

In this article, we introduce nQMaker to estimate nonreversible models from large data sets including hundreds to thousands of MSAs. We used nQMaker to estimate six nonreversible models: a general protein model from Pfam and five clade-specific data sets for birds, insects, mammals, plants, and yeasts, respectively. Our analyses show that the nonreversible models uncover distinct patterns of amino acid substitutions not captured by traditional reversible models, that the nonreversible models affect the inference of tree topologies, and allow for the estimation of root positions without outgroups.

Our results show that nonreversible models are more favorable to reversible models when increasing the size of the alignment. Nonreversible models were selected using standard model selection approaches for most single-locus alignments. In concatenated multilocus alignments, nonreversible models tended to be the best fit model in practically all data sets with at least 20 loci. The trees inferred with nonreversible models were often topologically different from those constructed with reversible models, suggesting that when a nonreversible model is the best-fit model for a data set, topological accuracy of phylogenetic inference may be improved.

Rooting phylogenetic trees is an essential task in studying evolutionary relationships among species. This is normally accomplished by using outgroup species or additional assumptions such as molecular clocks (Huelsenbeck et al. 2002). Nonreversible models provide an alternative approach that implicitly enables the reconstruction of rooted trees as part of the model. Our analyses of Bird and Plant data sets with nonreversible models identified the root of the trees of these groups with a very high-statistical confidence that agree with previous studies (Jarvis et al. 2015; Ran et al. 2018). Together with other encouraging results on mammals (Naser-Khdour et al. 2021) and from simulated data (Bettisworth and Stamatakis 2021), this provides increasing evidence that nonreversible models are effective in identifying root placements for empirical data sets, and will be useful when an appropriate outgroup is difficult to obtain.

The nonreversible models consist of 379 parameters describing all pairwise substitution rates between 20 amino acids. Therefore, they should be estimated from large data sets consisting of hundreds to thousands MSAs to avoid overfitting the training data. The six nonreversible rate matrices we estimate in this study are now available in the latest version of IQ-TREE 2, allowing researchers to easily use these models for their analyses. We recommend that users perform model selection to determine the best fit model for any specific alignment under study, and note that it is possible to combine both reversible and nonreversible models in a single partitioned analysis. The nQMaker algorithm is implemented in IQ-TREE 2, so researchers can estimate nonreversible models from their own data sets. For example, the NQ.plant model was estimated from 1000 plant alignments in 1.5 days using a computer with 36 cores.

A limitation of our models is that although relaxing the time reversibility, they still assume stationarity, that is, the amino acid frequencies stay constant along the tree. However, the stationary assumption is highly likely to be violated during the evolution of distantly related proteins, for example, between bacteria and eukaryotes. Failure to account for heterogeneous sequence composition might mislead phylogenetic reconstruction. Apart from nonstationary models, one can also use a mixture model of several Q matrices such as C10-C60, LG4M, and LG4X (Le et al. 2012). Deriving nonstationary and/or additional mixture amino acid models is an important avenue of future research.

## Supplementary material

Data available from the Dryad Digital Repository: https://doi.org/10.5061/dryad.3tx95x6hx.
