# Correction to: nQMaker: Estimating Time Nonreversible Amino Acid Substitution Models

**DOI:** 10.1093/sysbio/syag043

**Published:** 2026-06-10

This is a correction to: Cuong Cao Dang, Bui Quang Minh, Hanon McShea, Joanna Masel, Jennifer Eleanor James, Le Sy Vinh, Robert Lanfear, nQMaker: Estimating Time Nonreversible Amino Acid Substitution Models, *Systematic Biology*, Volume 71, Issue 5, September 2022, Pages 1110–1123, https://doi.org/10.1093/sysbio/syac007

The authors found an error in the PCA analysis underlying Figure 4, which arose from their use of matrix multiplication instead of element-wise multiplication during the conversion of the exchangeability matrix to the full instantaneous substitution rate matrix. This computational error affected the visualization shown in Figure 4 and, consequently, one associated interpretive statement in the main text.

This correction does not affect the performance results of the nonreversible models, nor does it alter any conclusions of the paper.

Figure 4 using the correct computation should read:

The corrected figure shows that each nonreversible model is positioned closely to its corresponding reversible model, rather than forming a distinct cluster as previously stated.

The affected statement in the body of the article (in section **Analysis of the Properties of Nonreversible Models**) is:

“Figure 4 illustrates the PCA analysis of 6 nonreversible models and 25 existing reversible models. Figure 4 shows that the models group into three distinct clusters, that is, one cluster of nonreversible models, one cluster of reversible models estimated from mitochondrial data, and another cluster of reversible models estimate from other genomic regions. This PCA analysis indicates that nonreversible models provided a very distinct pattern of amino acid substitutions not captured by existing reversible models.”

**Figure:**
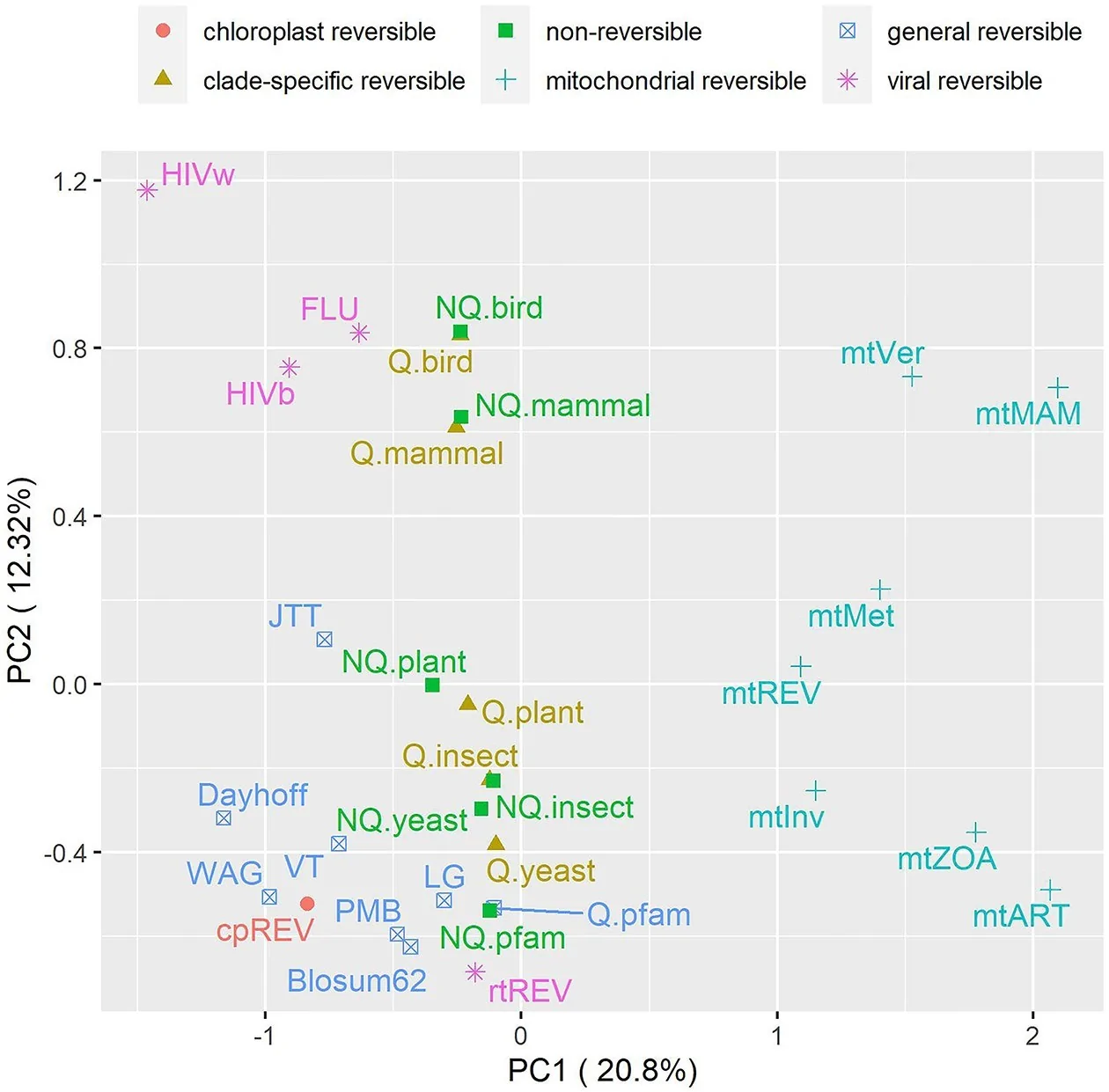


and this should read:

“Figure 4 presents the PCA analysis of six nonreversible models and 25 existing reversible models. The results show that each nonreversible model is positioned closely to its corresponding reversible model, indicating substantial similarity between their inferred amino acid substitution patterns. Reversible models estimated from mitochondrial data and those estimated from other genomic regions remain separated in the PCA space, while the associated nonreversible models align with their respective reversible counterparts.”

The errors, for which the authors take full responsibility, have been outlined only in this correction notice to preserve the version of record.

